# A Preliminary Trial in the Efficacy of Yokukansankachimpihange on REM Sleep Behavior Disorder in Dementia With Lewy Bodies

**DOI:** 10.3389/fnut.2020.00119

**Published:** 2020-08-14

**Authors:** Yuta Manabe

**Affiliations:** ^1^Department of Dementia and Geriatric Internal Medicine, Kanagawa Dental University, Yokosuka, Japan; ^2^Department of Emergency and General Internal Medicine, School of Medicine, Fujita Health University, Toyoake, Japan; ^3^Department of Internal Medicine, Dementia Diagnosis Center, Yokohama Shintoshi Neurosurgical Hospital, Yokohama, Japan

**Keywords:** clonazepam, Lewy body disease, polysomnography, REM sleep behavior disorder, traditional herbal medicine

## Abstract

**Background:** Clonazepam (CNZP) is effective in ~90% of patients with rapid eye movement sleep behavior disorder (RBD) but has risks of oversedation, muscular relaxation, and adverse effects on cognitive function when used to treat RBD associated with dementia with Lewy bodies (DLB). Yokukansankachimpihange (YKSCH), a traditional herbal medicine, decreases sleep latency and increases sleep stage 2, like benzodiazepines (BZPs), but does not cause adverse events such as oversedation, muscular relaxation, and adverse effects on cognitive function. Given these pharmacological properties, YKSCH was studied as a potential alternative to CNZP.

**Methods:** Of patients who were diagnosed with DLB according to the criteria for the clinical diagnosis of DLB established by the Consortium on Dementia with Lewy Bodies (CDLB) in 2017, 13 consecutive patients with the cutoff score (5 points) or more in a REM sleep behavior disorder screening questionnaire and polysomnographic evidence of REM without atonia were observed using the Neuropsychiatric Inventory (NPI) night-time behavior disturbance, visual analog scale (VAS) frequency, and VAS severity as the co-primary endpoints. Data from 11 patients who completed the study were statistically analyzed.

**Results:** Statistically significant improvements were observed in the NPI night-time behavior disturbance, VAS frequency, and VAS severity. No notable adverse events were reported.

**Conclusion:** The results indicated that YKSCH, which does not cause oversedation, muscular relaxation, or adverse effects on cognitive function, may provide a new therapeutic option for RBD associated with DLB as an alternative to CNZP.

## Introduction

Rapid eye movement sleep behavior disorder (REM sleep behavior disorder; RBD) is a parasomnia involving dream enactment behavior (DEB). Among neurodegenerative diseases, this disease attracts special interest because of its close relationship with α-synucleinopathy, including Lewy body diseases (LBD) such as Parkinson's disease (PD) and dementia with Lewy bodies (DLB), and multiple system atrophy (MSA), as evidenced by a report that half of patients with RBD develop PD-related disease within 10 years (1). Schenck et al. reported that ~38% of 29 patients with idiopathic RBD developed PD after a mean time of 3.7 years since diagnosis (2), with a 16 year follow-up report that RBD progressed to PD-related disease or dementia in ~81% of patients (3). In addition to these clinical studies, Boeve et al. conducted a neuropathological study in 172 patients with RBD and reported that 93% of the patients had α-synucleinopathy, including 136 patients with LBD (and Alzheimer's disease in 59 patients) (4). Based on many findings, including the aforementioned, the criteria for the clinical diagnosis of DLB in 2017 have raised RBD to a core feature of DLB and added polysomnographic (PSG) evidence of REM without atonia (RWA) as a new biomarker indicative of DLB (5).

In the treatment of RBD, clonazepam (CNZP) is effective in ~90% of patients with RBD (6), and it was reported that CNZP was as effective for RBD associated with PD as for idiopathic RBD, leading to the use of CNZP as the standard of care (7), although no randomized comparative study in LBD has been conducted to evaluate the usefulness of CNZP. While RBD, which poses a risk of injury to patients and their bed partners, requires aggressive therapeutic intervention, patients with LBD, especially DLB, are hypersensitive to drugs and vulnerable to the side effects of CNZP, including oversedation, muscular relaxation, and adverse effects on cognitive function. Hence, drugs that improve RBD by acting on the sleep architecture without muscular relaxation are awaited, and potential alternative treatments include Yokukansankachimpihange (YKSCH), a traditional herbal medicine, which has been reported to be effective for sleep disorder, including RBD associated with PD (8). To the best of our knowledge, however, the position of YKSCH in the treatment of sleep disorder, including RBD associated with DLB, has not been studied or reported. YKSCH, which decreases sleep latency and increases sleep stage 2 (9), is approved for the treatment of sleep disorder in Japan and is widely used in clinical settings, partly due to its large safety margin, a feature common to traditional herbal medicines.

We studied YKSCH as a potential alternative to CNZP for RBD associated with DLB based on our experience, previous reports, and pharmacological features of YKSCH.

## Methods

### Participants

Thirteen new patients who visited our hospitals from September 1, 2017, to March 31, 2019, and met all of the inclusion criteria listed below were included in the study.

### Inclusion and Exclusion Criteria

1. Patients who were diagnosed with DLB as evidenced by ^123^I-MIBG myocardial scintigraphy and/or ^123^I-ioflupane SPECT according to the criteria for the clinical diagnosis of DLB established by the Consortium on Dementia with Lewy Bodies (CDLB) in 2017. Patients who met the diagnostic criteria but were not diagnosed with dementia but had a Mini-Mental State Examination (MMSE) score of ≥24 were included as candidate subjects with pre-dementia stage of DLB.
2. Aforementioned candidate subjects who were definitively diagnosed with RBD based on the cut-off score (5 points) or more on the REM sleep behavior disorder screening questionnaire (REM sleep behavior disorder screening questionnaire—Japanese version; RBDSQ-J) and had polysomnographic (PSG; PSG-1100, Nihon Kohden Corp., Tokyo, Japan) evidence of RWA. The cut-off score was based on the results of a validation study of the RBDSQ-J conducted by Miyamoto et al. (10).
3. Patients who had not received YKSCH or Yokukansan of the same class, and they were drug-naïve about psychotropics.
4. Patients who met all of the aforementioned criteria and provided oral and written informed consent to participate in the study after receiving an explanation of the study.

Preexisting psychotropic drugs were allowed at stable doses, with dose modification prohibited throughout the observation period. Starting treatment with psychotropic drugs, including acetylcholinesterase inhibitors, anti-parkinsonian drugs, and hypnotics, was prohibited during the observation period.

Those who, in the opinion of the clinician, had disease or comorbidity inadequate for participation in the study such as severe cardiac failure, assisted-living residents, those living alone, and those who had no consistent bed partner were excluded from the study.

### Procedures

Thirteen patients meeting all of the aforementioned criteria orally received YKSCH at a dose of 3.75 g before dinner and at bedtime. Symptoms were assessed in terms of the endpoints described below before (Week 0) and 4 weeks after the start of treatment.

RBD was assessed by bed partners using the Neuropsychiatric Inventory (NPI) night-time behavior disturbance and the visual analog scale (VAS) for frequency and severity. The VAS for frequency was defined as follows: 0: neither talking nor shouting during sleep, nor intense movements of limbs are observed; 1: talking or shouting during sleep and/or intense movements of limbs are observed less than once a week; 2: talking or shouting during sleep and/or intense movements of limbs are observed at least once a week; and 3: talking or shouting during sleep and/or intense movements of limbs are observed every day. The VAS for severity was defined as follows: 0: neither talking nor shouting during sleep nor intense movements of limbs are observed; 1: talking or shouting during sleep and/or intense movements of limbs are observed but do not bother the bed partner; and 2: talking or shouting during sleep and/or intense movements of limbs are observed and bother the bed partner.

The cognitive function of subjects was assessed using the MMSE, the revised Hasegawa's dementia rating scale (HDS-R), and the Japanese version of Montreal Cognitive Assessment (MoCA-J). The motor symptoms were assessed using the Movement Disorder Society-Unified Parkinson's Disease Rating Scale Part III (MDS-UPDRS III).

In addition, adverse events, including hypokalemia, edema, and weakness, were monitored at Weeks 0 and 4 by means of general physical examination and blood biochemical tests.

### Statistical Analysis

Collected data were statistically analyzed using Wilcoxon's signed-rank test using the statistical analysis software EZR Version 1.37 (Easy R, The R Foundation for Statistical Computing, Vienna, Austria) (11).

This study was conducted after being reviewed and approved by the institutional review board of Yokohama Shintoshi Neurosurgical Hospital. The author declares no conflict of interest.

## Results

Of 13 subjects, 2 dropped out of the study; therefore, the remaining 11 subjects were included in the analysis. The reasons for dropout were as follows: one subject who was found to have breast cancer after informed consent withdrew consent to receive treatment for breast cancer, and the other subject voluntarily dropped out of the study due to poor treatment compliance.

**Figure 1:**
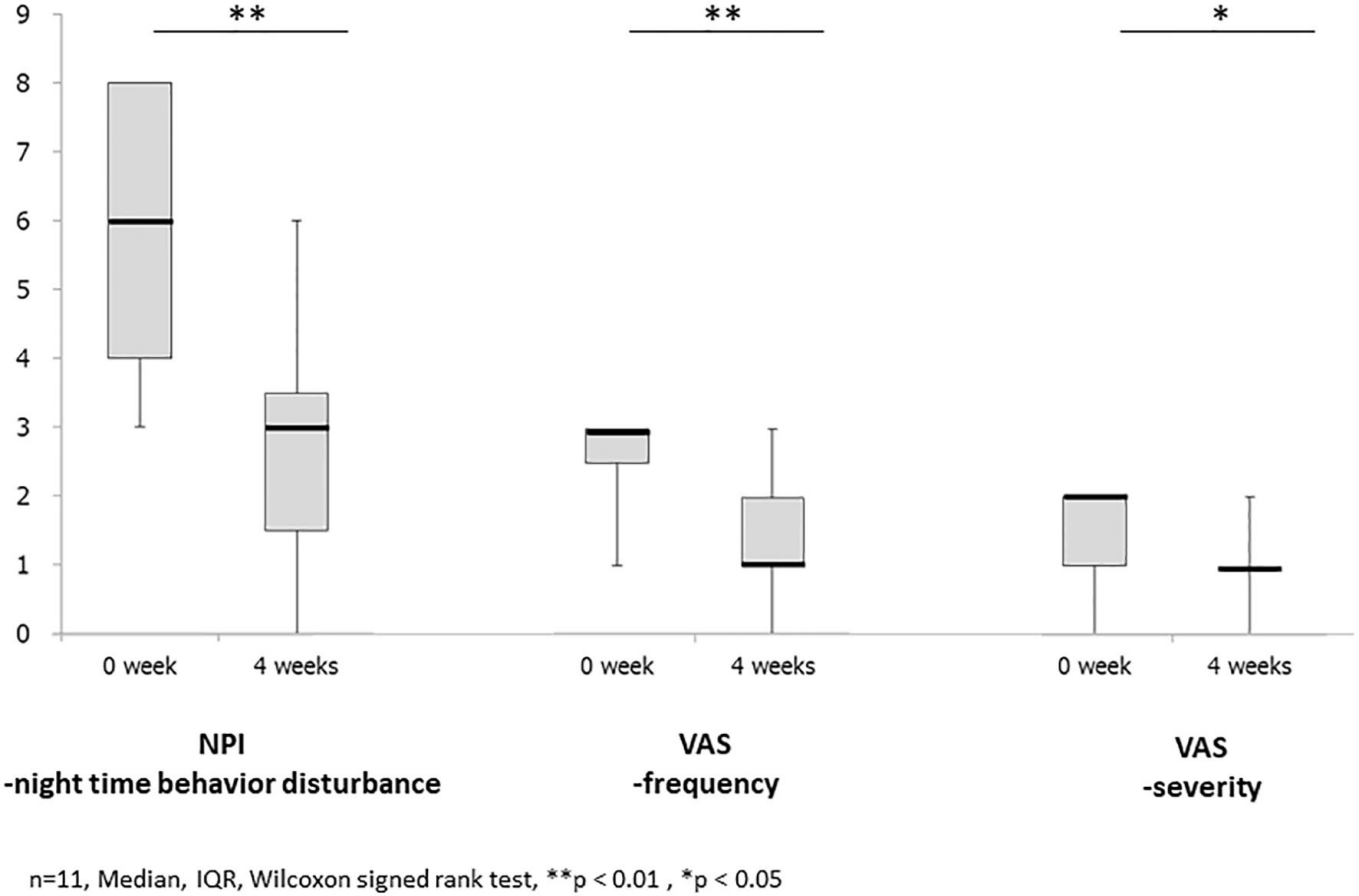
It shows the results of changes before and after treatment with Yokukansankachimpihange in statistical analysis. The mean and median scores improved in NPI night-time behavior disturbance, VAS, frequency, and severity with statistically significant differences observed between the two time points. There is no statistically significant difference in the serum potassium level.

Baseline data for individual subjects are presented in Table 1, and the changes after for weeks from baseline of NPI night-time behavior disturbance, VAS for frequency and severity, and serum potassium level after 4 weeks are shown in Table 2. Furthermore, the results of statistical analysis are presented in Figure 1.

**Table 1:** The baseline data for individual subjects.

**Sex**	**Age**	**Diagnosis**	**MMSE**	**HDS-R**	**MoCA-J**	**RBDSQ-J**	**MDS UPDRSIII**	**NPI-night time behavior disturbance**	**VAS frequency**	**VAS severity**	**Serum potassium (mEq/l)**	**Concomitant drugs**
1 M	85	DLB	23	24	18	6	1	8	3	2	4.7	
2 M	76	Pre-dementia stage of DLB	25	26	22	8	0	8	3	2	4	
3 M	66	Pre-dementia stage of DLB	30	29	26	8	1	4	3	2	4.6	
4 F	74	DLB	25	22	19	7	2	4	3	2	4.1	
5 M	81	DLB	24	21	17	9	6	8	3	2	4.5	Pitavastatin, Clopidogrel
6 M	76	Pre-dementia stage of DLB	26	26	25	5	0	4	2	1	4.5	
7 F	81	DLB	16	14	11	5	10	6	2	1	5.1	
8 M	71	Pre-dementia stage of DLB	30	28	24	6	0	3	1	1	4.2	Topiroxostat, Zinc
9 F	89	DLB	20	23	14	6	3	4	3	1	5.1	
10 M	66	Pre-dementia stage of DLB	30	30	27	8	0	8	3	2	4.7	
11 M	76	Pre-dementia stage of DLB	29	29	24	10	0	8	3	2	4.4	Telmisartan, Benidipine Atrorvastatin, Famotidine

*DLB, dementia with Lewy bodies; HDS-R, the revised Hasegawa's dementia rating scale; MoCA-J, Japanese version of Montreal Cognitive Assessment; MMSE, Mini-Mental State Examination; MDS-UPDRSIII, Movement Disorder Society- Unified Parkinson's Disease Rating Scale part III; NPI, Neuropsychiatric Inventory; VAS, visual analog scale*.

**Table 2:** The changes after 4 weeks from baseline of NPI night-time behavior disturbance, VAS for frequency and severity, and serum potassium level.

**Sex**	**Age**	**Diagnosis**	**NPI night time behavior disturbance**		**VAS frequency**		**VAS severity**		**Serum potassium (mEq/l)**	
			**0 w**	**4 w**	**0 w**	**4 w**	**0 w**	**4 w**	**0 w**	**4 w**
1 M	85	DLB	8	2	3	1	2	1	4.7	4.4
2 M	76	Pre-dementia stage of DLB	8	2	3	1	2	1	4	3.9
3 M	66	Pre-dementia stage of DLB	4	4	3	3	2	2	4.6	5.1
4 F	74	DLB	4	1	3	1	2	1	4.1	4
5 M	81	DLB	8	6	3	2	2	1	4.5	4.5
6 M	76	Pre-dementia stage of DLB	4	4	2	2	1	1	4.5	4.2
7 F	81	DLB	6	3	2	1	1	1	5.1	4.1
8 M	71	Pre-dementia stage of DLB	3	0	1	0	1	0	4.2	4.7
9 F	89	DLB	4	3	3	2	1	1	5.1	4.7
10 M	66	Pre-dementia stage of DLB	8	0	3	0	2	0	4.7	4
11 M	76	Pre-dementia stage of DLB	8	3	3	2	2	1	4.4	4.5

*DLB, dementia with Lewy bodies; NPI, Neuropsychiatric Inventory; VAS, visual analog scale*.

Of the 11 subjects, 8 were male, and 3 were female, with a mean age of 76.5 ± 2.3 years; 5 had DLB, and 6 had pre-dementia stage of DLB. All female subjects had DLB. These demographic characteristics of subjects are presented in Table 3.

**Table 3:** The demographic characteristics of subjects.

Total participants	11
Men	8
Women	3
Age	76.5 ± 2.3
The number of patients with DLB	5
The number of patients with pre-dementia stage of DLB	6
MMSE	25.3 ± 4.5 median: 25 (16–30)
HDS-R	24.7 ± 4.7 median: 26 (14–30)
MoCA-J	20.6 ± 5.2 median: 22 (11–27)
MDS-UPDRS III	2.1 ± 3.2 median: 1 (0–16)

*DLB, dementia with Lewy bodies; HDS-R, the revised Hasegawa's dementia rating scale; MoCA-J, Japanese version of Montreal Cognitive Assessment; MMSE, Mini-Mental State Examination; MDS-UPDRSIII, Movement Disorder Society- Unified Parkinson's Disease Rating Scale part III*.

**Figure 2:**
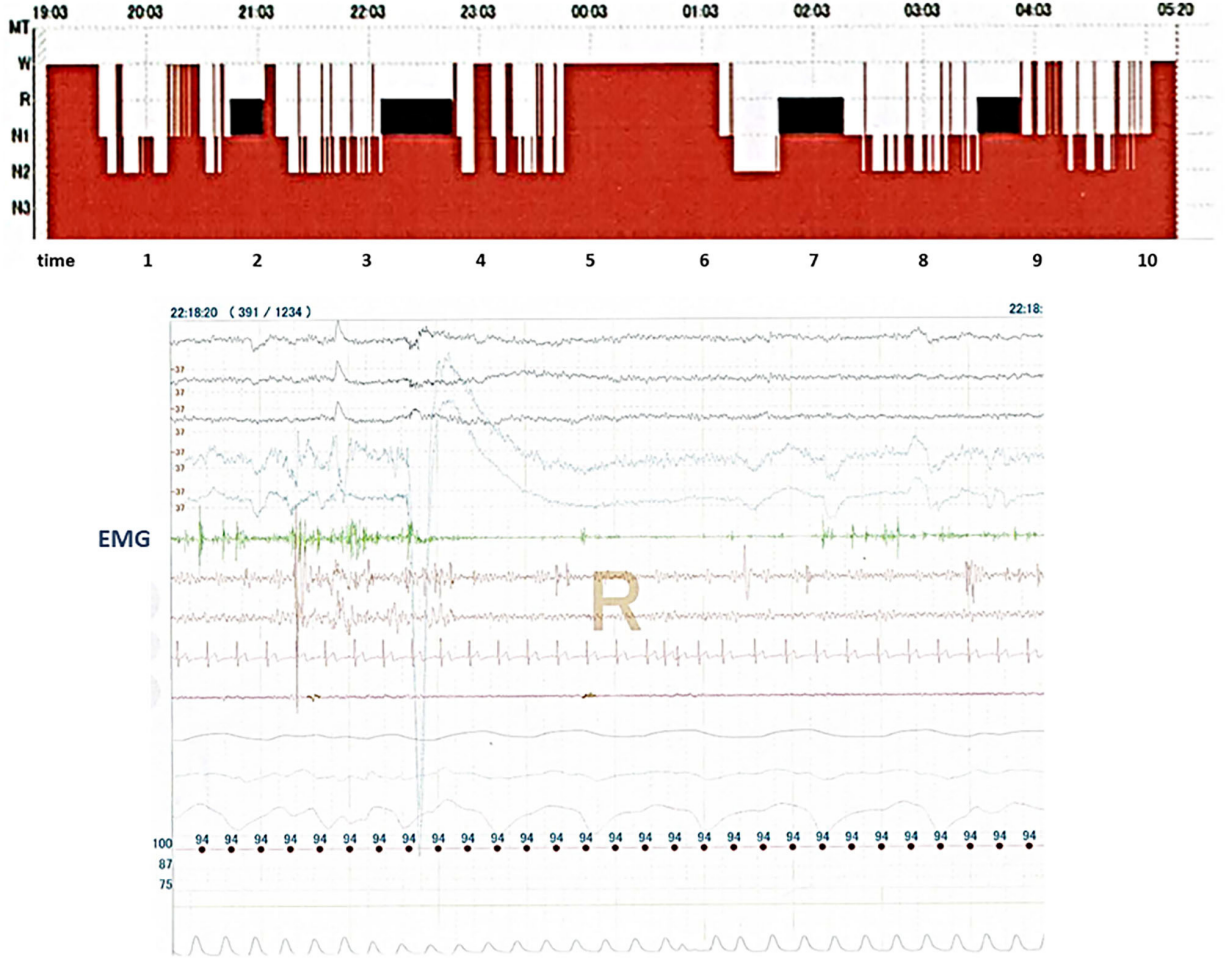
It shows records of polysomnography (PSG) in Case 11. The top part of the figure shows sleep stages in Case 11, and the bottom part of the figure shows records from many physiological signals including electroencephalograms, electrooculograms, electromyograms (EMG), nasal–oral flow, chest and abdominal movements, electrocardiograms, and arterial oxygen saturation which obtained PSG in Case 11. REM sleep without atonia is seen on the record of EMG channels during polysomnography.

On behalf of subjects, the polysomnogram and polysomnographic findings obtained from PSG in Case 11 are presented in Figure 2. The polysomnogram revealed REM sleep from 22:03 to 23:03, whereas the concurrent electromyogram (EMG) revealed RWA.

### NPI Night-Time Behavior Disturbance

The mean and median scores improved from 5.9 ± 2.1 and 6 (3–8) at baseline to 2.5 ± 1.8 and 3 (0–6) after treatment with YKSCH, respectively, with statistically significant differences observed between the two time points (*p* < 0.01) (Figure 1).

Individually, there were no changes in score from baseline after treatment with YKSCH in Cases 3 and 6.

### Visual Analog Scale—Frequency

The mean and median scores improved from 2.6 ± 0.7 and 3 (1–3) at baseline to 1.4 ± 0.9 and 1 (0–3) after treatment with YKSCH, respectively, with statistically significant differences observed between the two time points (*p* < 0.01) (Figure 1).

Individually, there were no changes in score from baseline after treatment with YKSCH in Cases 3 and 6.

### Visual Analog Scale—Severity

The mean and median scores improved from 1.6 ± 0.5 and 2 (1, 2) at baseline to 0.9 ± 0.5 and 1 (0–2) after treatment with YKSCH, respectively, with statistically significant differences observed between the two time points (*p* < 0.05) (Figure 1).

Individually, there were no changes in score from baseline after treatment with YKSCH in Cases 3 and 6.

### MDS-UPDRS III

There were no changes from mean score of 2.1 ± 3.2 or median score of 1.0 (0–10) at baseline after treatment with YKSCH; therefore, data were not statistically analyzed.

### Adverse Events

During the observation period, no serious adverse events, including edema, increased blood pressure, and acute cardiac failure, were reported. In addition, hearing of subjects or their bed partners revealed no episodes of fall associated with oversedation or muscular relaxation.

The mean serum potassium level was 4.5 ± 0.4 mEq/L at Week 0 before treatment with YKSCH and 4.4 ± 0.4 mEq/L at Week 4 after the start of treatment. There was no statistically significant decrease in serum K level from baseline after treatment with YKSCH (*p* = 0.260).

## Discussion

CNZP is the standard of care for RBD associated with LBD as well as for idiopathic RBD. On the other hand, many patients with DLB have fall and subsequent facture, as evidenced by a report that DLB had a greater risk of admission to hospital (or death) because of most commonly fall-related injuries than AD (12). For the treatment of RBD associated with DLB, therefore, drugs that improve symptoms without muscular relaxation are awaited. YKSCH, a traditional herbal medicine used in the treatment of sleep disorder, decreases sleep latency and increases the total duration of sleep and sleep stage 2 (9). These actions are based on pharmacological mechanisms similar to those of benzodiazepines (BZPs), which are widely used in the treatment of sleep disorder (13). In addition, YKSCH contains Citrus Unshiu Peel as equal to Chimpi as a constituent crude drug, and hesperidin, an ingredient of Citrus Unshiu Peel, is metabolized to hesperetin, which has anxiolytic effects through the serotonergic system (14). Angelica root, another constituent crude drug, has been demonstrated to act on the GABA receptor, as a partial agonist at the 5-HT1A receptor, and to downregulate the 5-HT2A receptor (15, 16), and has been reported to act on the BZP receptor to have anxiolytic effects (15). Geissoschizine methyl ether, which is contained in Uncaria hook, also acts as an agonist at the 5-HT1A receptor to have anxiolytic and antidepressant effects (17). Taken together, YKSCH, like CNZP, may alleviate the symptoms of RBD directly by acting as an agonist at the BZP binding site of the GABAA receptor, but the ingredients of various constituent crude drugs may also play a role in alleviating nightmare and DEB by acting on serotonergic neurons.

Regardless of the above, there are limits to discussion of potential mechanisms of action of YKSCH against RBD. More specifically, since REM sleep may be generated in the brain stem, lesions responsible for RBD are suspected to be damaged to neuronal nuclei involved in regulating REM sleep, including the locus coeruleus, pedunculopontine tegmental nucleus, and medullary gigantocellular reticular nucleus, but have not yet been identified; therefore, the reason why DEB occurs, that is, the mechanism underlying the development of RBD, is unknown. In addition, the mechanism of action of CNZP, the gold standard treatment for RBD, has not been elucidated, including whether CNZP produces radical cure by acting directly on the etiology of RBD or only alleviates symptoms by acting on the sleep architecture or reducing dream-induced anxiety. Since neither the pathophysiology of RBD nor the mechanism of action of CNZP is clear, the mechanism of action of YKSCH can be hypothesized or assumed but unfortunately cannot be determined. It is absolutely crucial to elucidate the mechanism underlying the development of RBD, including essential neuropathological findings. In addition, one of roles of REM sleep is memory retention. Therefore, it is necessary to consider what kind of influence occurs for a memory function by controlling REM sleep. Similarly, Matsui et al. pointed out in his paper about efficacy of Yokukansan (YKS) for the treatment of RBD (18). YKS is a herbal medicine with the same indications of YKSCH. By the way, the authors said that a mixture of various ingredients derived from seven medical herbs in YKS makes it more difficult to identify the specific positive mechanism of action for RBD symptoms in that paper. However, the glutamate uptake function of YKS suggests that the drug possibly reduces oneiric behavior through the suppression of phasic muscle activity as one of hypothesis with a possibility.

While the pathophysiology of RBD and the mechanism of action of CNZP remain unclear, the present study demonstrated that YKSCH improved RBD associated with DLB without causing fall due to oversedation or muscular relaxation or impairing cognitive function, indicating that YKSCH may be a potential alternative to CNZP. In addition, since patients with a definitive diagnosis of RBD as determined by PSG were enrolled in the study, the usefulness of YKSCH in the treatment of RBD was evaluated in subjects with an accurate diagnosis. This is particularly important in evaluation of the potential of YKSCH for RBD.

Finally, I discuss two cases that did not respond to a treatment and several limitations in this study.

The total number of pre-dementia stage of DLB was six cases and all patients were male. Two cases of those, Case 3 and Case 6, were not improved in clinical measures by using YKSCH. They had no significant differences in age, sex, level of cognitive function, and MDS-UPDRS III, among others. In an elemental analysis of PSG, Arousal Index (AI: awakening more than 3 s per 1 h) was 20.7% in Case 3 and 6.4% in Case 6. The mean AI was 15.8% in total subjects with pre-dementia stage of DLB. Hence, a low or high percentage of AI cannot be the cause of ineffective results.

One of possible causes is a ratio of REM sleep during the sleep (REM%). The mean REM% was 17.4% in total subjects, and the mean REM% in cases except Cases 3 and 6 was 24.9%. By contrast, REM% of Case 3 was 5.0 and 13.2% in Case 6. REM% in these two cases showed a significantly low frequency of REM sleep during the total sleep. One of the mechanisms of YKSCH against RBD is the increase in the total duration of sleep and sleep stage 2; therefore, REM sleep is relatively decreased. The patients had a few REM% in the first place; thereby, it is suggested that YKSCH may be ineffective against RBD in these two cases.

Second, since the subjects had DLB, the NPI night-time behavior disturbance was used as the measure of RBD in view of behavioral and psychological symptoms of dementia. The Parkinson's Disease Sleep Scale (PDSS), the recommended scale for sleep disorder associated with PD, was not employed in the study. In view of the neuropathological connection between PD and the target disease, including pre-dementia stage of DLB, however, the PDSS perhaps should have been selected as another assessment tool, although the disease of subjects was not pure PD.

To determine the pathophysiology of RBD or the pharmacological mechanisms of YKSCH, furthermore, PSG should be used to investigate how YKSCH changes the onset of RWA and the sleep architecture itself, including REM sleep, and whether YKSCH treats RBD fundamentally. Since the present study was conducted under the regulations of Japanese health insurance law, PSG could not be used after therapeutic intervention because of medical economic or legal constraints. Furthermore, because of the strict inclusion and exclusion criteria and the shortness of the entry period, the sample size in this study became small. These factors might carry a high risk of causing false-positive results. Therefore, the results in this study should be regarded as reference value.

Hence, the aforementioned problems should be addressed in future studies. With the limitations due to the preliminary nature of the current study in mind, we would like to collect further evidence.

## Conclusion

The potential of YKSCH, which, like CNZP, acts on the sleep architecture but does not cause oversedation or muscular relaxation, was studied for the treatment of RBD associated with DLB. The results of this study, although there were limitations due to the preliminary nature of the study, verified the hypothesis, indicating that YKSCH may provide a new therapeutic option for RBD associated with DLB.

## Data Availability Statement

All datasets generated for this study are included in the article/supplementary material.

## Ethics Statement

The studies involving human participants were reviewed and approved by the institutional review board of Yokohama Shintoshi Neurosurgical Hospital. The patients/participants provided their written informed consent to participate in this study.

## Author Contributions

YM made the conception or design of this work, the acquisition, analysis, interpretation of data for this study, described draft of this study, revised this manuscript, decided final approval of the version to be published, and will be responsible for that to the end.

## Conflict of Interest

The author declares that the research was conducted in the absence of any commercial or financial relationships that could be construed as a potential conflict of interest.
